# Dislocations of the Clavical

**Published:** 1855-12

**Authors:** Dan. V. Folts

**Affiliations:** 38 Maverick Square, Boston


*Dislocations of the Clavical.* Communicated for the Boston Medical and Surgical Journal.

From its anatomical relations and connections, the clavicle is more frequently the subject of accident than any other bone in the human skeleton. It was rightly named "clavis," not only from its resemblance to the ancient key, but as being the key itself that has unlocked the fortunes or misfortunes of many a chirurgical aspirant. Fractures of the clavicle are of far more frequent occurrence than the accident under consideration; the proportion, according to some of our best authorities, being as six to one. It is not long since that I was called to visit a boy of some dozen years, who the messenger told me, had fallen and injured his shoulder. I found fracture of the collar bone; and, after applying my dressings, a younger brother was brought in, and an examination discovered the same accident, only on the opposite side! But dislocations of the bone I have never found quite so common All surgical writers agree that the clavticle may be displaced at both its proximal and distal extremities. But the proportion in which these luxations occur, the indications for the restoration of the parts, and the means to be employed for fulfilling these indications, are items not so well established—are points upon which surgeons are not so well agreed. Boyer, Desault and Samuel Cooper represent that the sternal end of the bone is most frequently displaced; while Sir A. Cooper and other writers have found the scapular extremity most frequently dislocated. Of the 9 cases reported by Professor Hamilton,* 8 occurred at the scapular

*Transactions of the Medical Society of the State of New York, Albany, 1855.

and but one at the sternal extremity of the clavicle. Some authors speak of three dislocations of the sternal extremity, whilst others mention but two. Again, whilst some surgeons, of high authority, labor to build up an anatomical argument against the possibility, even, of the downward dislocation in scapular displacements; others, equally eminent in the profession, report cases of this very accident! But leaving these controverted points of minor consequence, let us at once apply ourselves to the practical part of our subject.

Can dislocations of the clavicle be reduced, and coaptation be so perfectly maintained, that no deformity shall result? The highest authorities on both sides of the Atlantic say no. Sir A. Cooper was accustomed, at the conclusion of his lecture on this subject, to say to his class, "You are not to expect that the parts, after the utmost care in the treatment, will, in dislocations of either end of the clavicle, be exactly adjusted. Some projection, some slight deformity, will remain." Samuel Cooper, in his Surgical Dictionary, affirms that "the exact maintenance of the reduction by any apparatus whatever, is found to be a matter of the greatest difficulty, and some deformity will remain." Dr. Reese, however, in his edition of the work, says in a note that "Dr. Cook, of Baltimore, has reported a case of the successful reduction of a dislocation of the clavicle at its scapular articulation."

M. Boyer remarks, that "notwithstanding the greatest assiduity of the surgeon, the luxated extremity will remain more prominent than that of the opposite side"—and that "this inevitable deformity would not be prevented, even though the tourniquet proposed by Brasdor were used." And Dr. F. Hamilton, in the Report already referred to, says, "I am quite sure that it will not be found often, if ever, practicable to retain the scapular end of the clavicle in place when it has been once dislocated; and the same difficulty will generally exist when the dislocation is at the sternal end. Of his 9 cases he remarked that "the clavicle was generally easily reduced, but in no instance was it made to remain in place." Mr. Ferguson, in his Practical Surgery, after giving directions how "an attempt may be made to keep the end of the bone (acromial) in its proper position," and assuring his readers that these means will in all likelihood not have the desired effect, concludes thus: "But unless the displacement be considerable, I believe it will be the best plan to let the shoulder alone, and merely keep the fore-arm in a sling." And of the sternal dislocation, he speaks in this wise: "Here, also, it is extremely difficult to keep the extremity in its proper seat—a false joint will in all probability be the result." And all this is endorsed by Dr. Norris, of the Pennsylvania Hospital, as good practice! I say endorsed—for he publishes Dr. F.'s article on dislocations of the clavicle, without comment. Mr. Liston and other writers hold much the same opinions, and all perfectly agree in looking upon the accident, if not quite, as pretty much beyond the resources of our art.

Here, then, is a mass of authority, and it might be very much increased, at once overwhelming, and to many surgeons entirely satisfactory. But what though M. Boyer and Desault, the Coopers and the Bells, were accustomed to be listened to as speaking *ex cathedra;* does it follow, as a matter of course, that their dogmas in this instance are correct? And henceforth must every dislocation of the clavicle, end, if not in the "formation of a false joint," at least in impaired function and "inevitable deformity"? However consoling it may be to the surgeon who has an unsuccessful case on his hands, to be able to bring all this array of authority to justify the result of his case; to the patient who had a lame limb, as his inheritance for life, an aching joint that is a perfect barometer, in every change of the atmosphere, it is a very different affair. But let me not be understood as censuring the opinions or condemning the practice of others in these remarks. They have with candor expressed their opinions, and given us the results of their practice. They have not covered up their cases because they were unsuccessful, and by their manly frankness have laid the professions under obligations for their contributions. I have been deeply interested in Dr. Hamilton's Report, but am not a subscriber to his articles of belief. I would not be understood as saying that every dislocation of the clavicle can, by any means whatever, be perfectly restored—or that three cases out of every four, even, can be successfully treated. But that it is an accident that does not always, of necessity, leave the joint deformed in its appearance and impeded in its functions, the following cases will most satisfactorily prove.

Case 1.—This was a dislocation of the scapular extremity of the clavicle, and occurred to a man in middle life, very athletic, and of as powerful muscular developments as I remember ever to have seen. In this case I did not find that it was "as easy to restore the bone to its proper connections, as it was difficult to retain it." The clavicle was found riding upon the spine of the scapula, and by the action of unusually developed muscles, the articular surfaces were widely drawn asunder.—Ether and cloroform were not then used, and it was only after persevering efforts that I succeeded in reducing the dislocation. Sir A. Cooper's clavicle bandage was applied, a cushion having been placed in the axilla—the shoulder was elevated as well as carried backward—the arm was secured to the side, and the fore-arm was suspended in a sling. A moderately thick compress was now placed over tho end of the dislocated bone and acromion, and this was well secured by a leather strap and buckle passing over the compress and under the point of the elbow. Thrice in the three following days was the bone partially thrown from its articular connections, but was much more easily reduced than at first; and was, after this period, so perfectly retained in situ, that my patient, in a short time, recovered entirely the use ot his shoulder, having no deformity whatever.

Case 2.—The subject of this accident was almost a fac-simile of the preceding—middle aged, strong, muscular. But the dislocation was at the sternal extremity of the bone. It occurred about two years after the first, and shortly after the introduction of ether. The dislocation was forward and upward in this case. Ineffectual attempts were made at reduction until the patient was fully etherized, when it was easily accomplished. The clavicular bandage was applied, and a compress retained over the dislocated extremity of the bone by turns of the roller. This succeeded for the day, but during the night the dislocation returned. Chloroform was again administered, and the bone restored to its normal connection. The same dressings were applied, with the addition of a splint so curved as to fit the outline of the chest and clavicle anteriorly, and make pressure on the sternal end of that bone, from before backward from above downward. This, well padded, was secured by casts of the roller, and answered the purpose tolerably well. The bone was retained in its place, and a pretty good cure effected. The only thing perceptible was an unnatural fulness of the joint; its functions, after a few months, being not materially impaired.

Case 3.—This was a sternal dislocation, the clavicle being thrown forward. It ocurred to a young man of about 25, on new year's eve, 1855. He was celebrating the holiday, when, unfortunately for himself, he took in too much ballast, considering the icy condition of our side-walks, and falling upon his shoulder he dislocated the collar bone. Having been summoned soon after the accident, I found not the slightest difficulty in reducing the dislocation without the use of ether. The patient had taken the alcohol and water uncombined with sulphuric acid, and it answered every purpose! Dr. Fox's apparatus, modifid and improved by Dr. E. Bartlett, of this city, was applied. This was the only dressing used, and the cure was perfect. There remained no deformity whatever, nor any impediment in the free use of the joint.

Case 4.—January 24th, 1855, a lad of 11 years was thrown violently upon the ice by a larger boy, dislocating the clavicle at its scapular extremity. Ether was administered, and the clavicular surfaces brought into exact coaptation. Dr. Bartlett's apparatus was applied, together with the compress and bandage over the acromion ; but it failed, as then constructed, of securing the end in view. A collar was placed around the shoulder of the affected side, and a strap passed from this to the apparatus on the sound shoulder, by means of which the scapulæ were approximated with a great degree of certainty, and so retained. The other indications were admirably fulfilled by the apparatus. A compress was now applied over the dislocated extremity of the bone, and firmly secured by the roller around the elbow. From this time forward there was no more difficulty. The parts were perfectly retained, and a speedy cure was effected ; the dressings being all removed February 24th. So entirely successful is this case in its results, that not the slightest trace of deformity can be seen ; and the most skilful surgeon cannot, by the most careful examination, tell me which clavicle was dislocated eight months ago.

Before dismissing this case, I would say that Dr. Bartlett's apparatus was of essential service in its successful treatment. And with the modifications he has since introduced, I consider it the most perfect, for the treatment of all injuries the clavicle is liable to sustain, of anything with which I am acquainted. It was intended for fractures of the clavicle only, but will answer well for dislocations of the sternal extremity, and must be considered a great acquisition in the treatment of acromial displacements. Let it not be forgotten, however, that the compress over the dislocated extremity of the clavicle, well secured by successive turns of the roller around the apex of the elbow, I consider a *sine qua non* in the successful treatment of the latter class of luxations.

It is at this point, in my opinion, that surgeons have failed. Sir A. Cooper speaks of having the straps of his clavicular bandage "wide enough to press upon the clavicle, scapula and os humeri." And Sir Charles Bell, in his Operative Surgery, points to "the spica bandage around the shoulder-joint," as the means by which the dislocated extremity of the bone is to be retained in its socket. But neither can be relied on, according to the testimony of these very authors themselves ; both will as surely disappoint the hopes of the surgeon as if he had applied, for his dressing, the shawl recommended by Liston ; or, with Mr. Ferguson, had merely suspended the fore-arm in a sling. It matters not how accurately the dislocation may be reduced, or how securely the scapula may be drawn backward and elevated, and the humerus fixed to the side ; if the action of that part of the trapezius implanted into the outer third of the clavicle be not contracted. For the serratus magnus, the pectoral and other muscles, do not, with more certainty, displace the scapula, than does this portion of the trapezius the scapular end of the clavicle when once dislocated. But the scapula and humerus being secured as above indicated, the apex of the elbow is, for all practical purposes, a fixed point, and one that is available in this accident. It affords us the means of completing a dressing at once efficient, and that in most cases of dislocations of the acromial articulation, must be finally successful. DAN. V. FOLTS, M. D.

38 Maverick Square, Boston, Oct., 1855.

